# PRG‐1 Relieves Neonatal Stimuli‐Induced Hyperalgesia and Anxiety via Stage‐Specific Synapse Remodeling

**DOI:** 10.1111/cns.70560

**Published:** 2025-08-18

**Authors:** Wenyu Zhang, Yan Li, Guangda Liang, Qingmei Li, Zhouyi Song, Song Cao, Zhi Xiao, Xingfeng Liu

**Affiliations:** ^1^ Guizhou Key Laboratory of Brain Science/Key Laboratory of Anesthesia and Organ Protection of Ministry of Education (in Cultivation) Zunyi Medical University Zunyi China; ^2^ Department of Anesthesiology Dejiang People's Hospital Tongren China; ^3^ Department of Pain Medicine The Tenth Affiliated Hospital of Southern Medical University (Dongguan People's Hospital) Dongguan China

**Keywords:** pain, plasticity‐related gene 1, repetitive noxious stimulation, synaptic remodeling, synaptic transmission

## Abstract

**Objective:**

Neonatal repetitive noxious stimuli (RNS), to mimic early‐life repetitive pain exposure, induce persistent hyperalgesia, anxiety‐like behaviors and postoperative pain sensitization that endure into adulthood. These long‐term neurobehavioral abnormalities are associated with impaired cognitive, emotional, and psychosocial functions.

**Method:**

We established a neonatal RNS rat model through repetitive needle pricks to all four limbs of neonatal rats and investigated the effects of hippocampal PRG‐1 and synaptic remodeling at different stages in RNS rat.

**Results:**

Our study demonstrates that hippocampal PRG‐1 dynamically modulates RNS‐induced hyperalgesia and anxiety through stage‐specific regulation of AMPAR GluR1/GluR2 and NMDAR GluN2A/GluN2B trafficking, which leads to synaptic remodeling via altered dendritic synaptic morphology and synaptic transmission efficacy.

**Conclusion:**

Our findings suggest that PRG‐1 relieves RNS‐induced persistent hyperalgesia, anxiety, and pain‐perception memory via synapse remodeling at different stages. Targeting PRG‐1‐mediated synaptic remodeling may provide a novel neuroprotective strategy for preventing chronic pain comorbidities with anxiety disorders following early‐life pain exposure.

## Graphical abstract

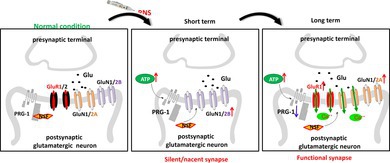

**Abbreviations**
ACC: anterior cingulate cortex
AMPAR: AMPA receptor, α‐amino‐3‐hydroxy‐5‐methyl‐4‐isoxazole propionate receptor
ATP: adenosine 5′‐triphosphate disodium salt solution
ATP: adenosine triphosphate
ATPγS: adenosine 5′‐[γ‐thio] triphosphate tetralithium salt
Aβ: β‐amyloid
BCP: bone cancer pain
BSA: bovine serum albumin
CBD: calmodulin‐binding domain
CCI: chronic constriction injury
CI‐AMPARs: Ca^2+^ impermeable AMPARs
CNS: central nervous system
CoIP: co‐immunoprecipitation
CON: control
CP‐AMPARs: Ca^2+^ permeable AMPARs
d.a.l.: day after lesion
DG: dentate gyri
DIV: days in vitro
E: embryonic day
ELISA: enzyme‐linked immunosorbent assay
EPM: elevated plus maze
ERG1: epilepsy related gene
F‐actin: actin filament
FST: forced swimming test
GA: glutaraldehyde
Glu: glutamate
GluR2: AMPA‐type glutamate receptor subunit 2
HRPO: horseradish peroxidase
I.D.: inner diameter
i.p.: intraperitoneal
IASP: International Association for the Study of Pain
IBS: irritable bowel syndrome
IF: immunofluorescence
IN: incision
KO: knock out
LPA: lysophosphatidic acid
LPP: lipid phosphate phosphatase
LPPRs: lipid phosphate phosphatase‐related proteins
LTP: long‐term potentiation
moDG: molecular layer of dentate gyri
MWT: mechanical withdrawal threshold
NICU: neonatal intensive care unit
NMDAR: NMDA receptor, N‐methyl‐D‐aspartate receptor
NS: normal saline
NSF: N‐ethylmaleimide sensitive fusion protein
O.D.: outside diameter
OE: overexpression
OFT: open field test
P: postnatal day
PFA: paraformaldehyde
PP2A: protein phosphatase 2A
PRG‐1: plasticity‐related gene 1
PRGs: plasticity‐related genes
RNS: repetitive noxious stimulation
SEM: standard error of mean
sEPSC: spontaneous excitatory postsynaptic potential
SI: silence
so: stratum oriens
sr: stratum radiatum
TWL: thermal withdrawal latency
WB: western blot
WT: wild type

## 1 Introduction

Neonates in the neonatal intensive care unit (NICU) endure repetitive noxious stimuli (RNS) averaging up to 14 times per day [1]. NICU experience or surgery is related to increased risks of neurodevelopmental delay, as well as changed maladaptive somatosensory function and pain sensitivity in adulthood [2]. Notably, up to a quarter of preterm infants develop internalizing behavioral disorders characterized by anxiety and depression trajectories in childhood or adulthood, manifesting as 2–6‐fold higher incidence compared to full‐term cohorts [3, 4], and may lead to higher rates of comorbid mental health, social, motor, and cognitive problems [5].

Newborn infants exhibit robust nociceptive responses to repetitive noxious stimuli [6], which, in the absence of analgesia, can induce maladaptive structural and functional remodeling of the developing nervous system, culminating in long‐term neuroplastic changes [1, 7, 8]. Neonatal surgical interventions alter somatosensory processing, leading to increased analgesic requirements, higher recurrence rates of postoperative pain, and amplified pain intensity during adulthood reoperations [2]. These findings support the notion that early‐life nociceptive exposure establishes enduring “somatosensory memory” traces [9, 10]. Upon subsequent nociceptive challenges, latent juvenile pain memories are reactivated, resulting in hyperalgesic responses that exceed those observed in individuals without early pain histories—a phenomenon implicated in persistent hyperalgesia, pathological pain memory formation, and other chronic pain sequelae. Notably, RNS induced pain effects exhibit greater longevity compared to other chronic pain modalities, underscoring their unique role in shaping neurodevelopmental trajectories of pain hypersensitivity.

These developmental vulnerabilities may originate from maladaptive synaptic reorganization triggered by unmitigated nociceptive input during critical neurodevelopmental windows. The fMRI studies showed that three brain regions (bilateral auditory cortex, hippocampus and caudate) are only active in infants experiencing pain but not in adults [11], suggesting the unique role of the hippocampus in neonatal pain stimulation. Plasticity‐related genes 1 (PRG‐1/LPPR4), an evolutionarily conserved lipid phosphate phosphatase predominantly expressed in the hippocampal primordium and differentially regulated during brain development [12, 13], play an important role in neurodevelopment, synaptic plasticity, and repair following injury [14, 15]. Our previous work reported that PRG‐1 relieves pain and depressive‐like behaviors in rats with bone cancer pain (BCP) by regulation of dendritic spine in the hippocampus [16]. Our recent findings implicate that PRG‐1 also plays a role in RNS‐induced pain [17]. However, the molecular mechanisms underlying PRG‐1‐mediated protection against maladaptive pain memory formation remain elusive.

A significant population of “silent synapses” exists in the brain and can be converted into functionally active synapses under specific physiological or pathological conditions. This conversion underpins synaptic plasticity mechanisms critical for learning and memory formation and their long‐term maintenance [18]. Emerging evidence further suggests that neuropathological conditions, including addiction, seizures, and chronic pain [19], promote aberrant synaptic transmission and maladaptive plasticity, resulting in silent synapse formation [20]. However, the regulatory mechanisms governing silent synapse dynamics during pain pathogenesis, particularly under RNS, remain poorly understood.

We established a neonatal RNS rat model to mimic early‐life repetitive pain exposure. Neonatal RNS induced persistent hyperalgesia, anxiety‐like behaviors, and sensitization of postoperative pain from childhood to adulthood. Hippocampal PRG‐1 not only prevented aberrant synaptic activity but also restored nociceptive processing and emotional dysfunction in RNS rats. Mechanistically, PRG‐1 dynamically regulated AMPAR GluR1/GluR2 and NMDAR GluN2A/GluN2B trafficking, which leads to synaptic remodeling through neuronal activity, synaptic morphology, synaptic firing properties, and neurotransmission efficacy. These findings suggest that PRG‐1 relieves RNS‐induced hyperalgesia, anxiety, and pain‐perception memory via stage‐specific synapse remodeling. PRG‐1 provides a novel pharmacological approach to prevent maladaptive nociceptive processing and confer neuroprotection in RNS. This study provides a foundation for exploring PRG‐1‐based interventions to mitigate long‐term neurodevelopmental consequences of early‐life pain in human infants.

## 2 Results

### 2.1 RNS Induces Persistent Pain, Anxiety‐Like Behaviors, and Pain Memory in Rats

**FIGURE 1:**
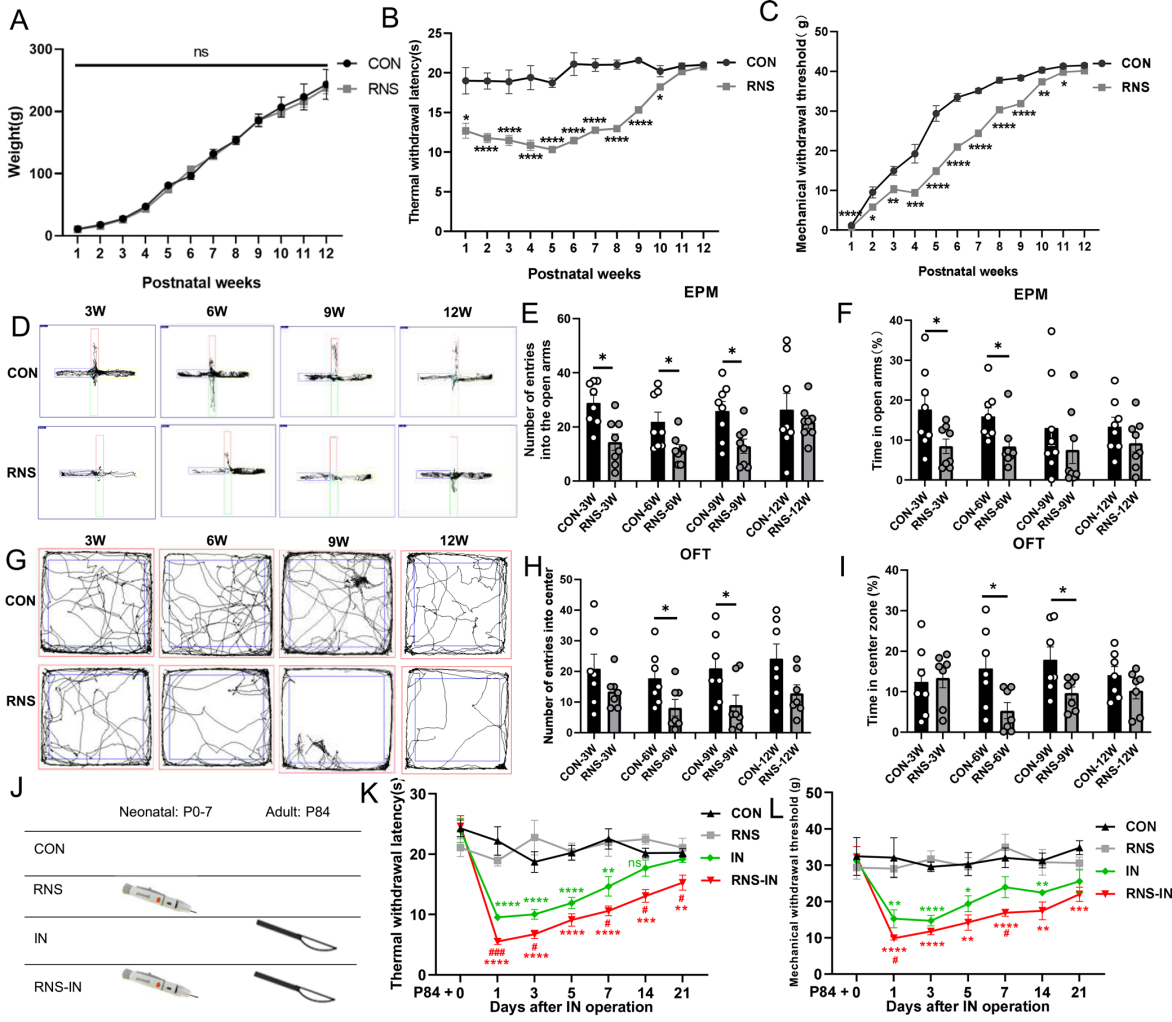
Neonatal RNS rats caused pain, anxiety‐like behaviors, and pain memory. (A) RNS procedure had no effect on the body weight of the rats from birth to adulthood (0–12 weeks) (*n* = 8; two‐tailed unpaired student *t*‐test, *p* > 0.05). (B, C) The TWL (B) and MWT (C), continuously monitored every week, were significantly decreased in RNS rats compared with age‐matched controls (*n* = 8, *: vs. CON group at the same time point; two‐tailed unpaired student *t*‐test). (D–F) The trajectory (D), number of times entering the open arm (E) and the percentage of time spent in the open arm (F) of rats in the elevated plus maze experiment (*n* = 8, *: vs. CON group at the same time point; E: Two‐tailed unpaired student *t*‐test, F: Mann–Whitney‐*U* test). (G–I) The trajectory (G), number of times into the central area (H) and the percentage of time spent in the central area (I) of rats in the open field test (*n* = 7, *: vs. CON group at the same time point; Mann–Whitney‐*U* test). (J) Diagram showing experimental groups to mimic the RNS‐induced exaggeration of adult incisional pain. (i) CON: Control adult rats. (ii) RNS: Adult rats having only RNS in the neonatal period. (iii) IN: Rats having only adult incision. (iv) RNS‐IN: Rats having neonatal RNS and adult incision. (K–L) RNS‐IN rats decreased TWL (K) and MWT (L) from Days 1 to 21 after adult incision (*n* = 6, *: vs. CON group at the same time point; #: vs. IN group at the same time point; two‐way ANOVA with Tukey's post hoc multiple comparisons test). Data are presented as mean ± SEM with n.s. *p* > 0.05; *p < 0.05; **p < 0.01; ****p < 0.0001. EPM, elevated plus maze; IN: incision; MWT, mechanical withdrawal threshold; OFT, open field test; RNS, repetitive noxious stimuli; TWL, thermal withdrawal latency.

RNS did not alter body weight across development (0–12 weeks; Figure 1A). To assess behavioral response to neonatal RNS, thermal withdrawal latency (TWL) and mechanical withdrawal threshold (MWT) were continuously monitored weekly in the rats from postnatal Week 1 to Week 12. TWL remained relatively stable from infancy to adulthood, while MWT increased gradually and stabilized in adulthood in CON rats, which is in line with what has previously been reported [2, 21, 22]. Compared to contemporaneous CON, neonatal RNS induced persistent thermal hyperalgesia and mechanical allodynia in rats, as evidenced by significant reductions in TWL and MWT from postnatal Week 1 to Week 10 (Figure 1B,C). In addition, TWL of RNS rats remained at a relatively stable low level from postnatal Week 1 to Week 8, while rising significantly from Week 9 and gradually approaching the TWL value of CON rats. Therefore, we will focus on what actually happened at Week 9.

Chronic pain and anxiety often affect each other and worsen each other. Elevated plus maze (EPM) and open field test (OFT) were used to evaluate the emotional changes in rats at different stages (Weeks 3, 6, 9, and 12). RNS rats exhibited anxiety‐like behaviors, characterized by reduced open‐arm entries and time spent in open arms (EPM; Figure 1D–F) and decreased entries and time spent in the central area (OFT; Figure 1G–I), persisting until postnatal Week 9. However, no dominance effect of RNS on pain or anxiety‐like behaviors was observed at Week 12, prompting investigation into the stealth effect of RNS by pain memory.

To model postoperative pain exacerbation by early‐life RNS, adult rats underwent incision surgery (RNS‐IN group) at Week 12 (Figure 1J). RNS‐IN rats exhibited heightened mechanical allodynia and thermal hyperalgesia from postoperative Day 1 to Day 21 compared to CON, RNS‐only, and incision‐only (IN) groups (Figure 1K,L). This priming effect indicates that neonatal RNS establishes latent sensitization, amplifying postoperative pain through maladaptive pain memory mechanisms.

### 2.2 Hippocampal PRG‐1 Alleviates RNS Induced Pain Hypersensitivity and Anxiety‐Like Behaviors

**FIGURE 2:**
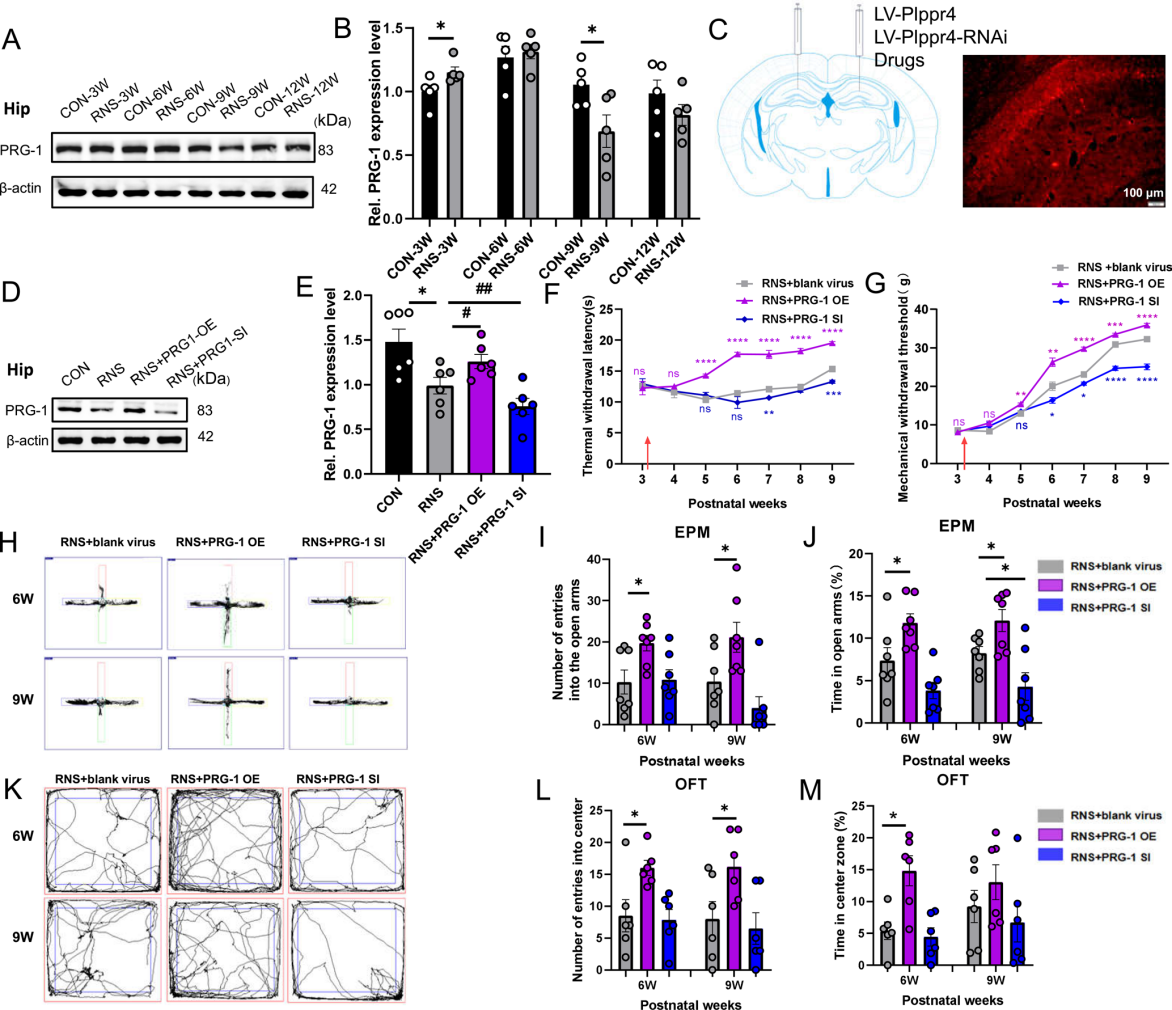
Hippocampal PRG‐1 alleviated RNS‐induced pain and anxiety‐like behaviors. (A) Western blot and (B) quantitative analysis of gray values (normalized to CON group of 3 weeks) revealed that PRG‐1 expression in the hippocampus of RNS rats was higher than in the age‐matched CON group at 3 W and decreased at 9 W (*n* = 5, *: vs. corresponding CON group, 3 W: Mann–Whitney‐*U* test; 6–12 W: Two‐tailed unpaired student *t*‐test). (C) Schematic diagram of hippocampal microinjection in the rat models. (D) Western blot and (E) quantitative analysis of gray values (normalized to CON values) showing PRG‐1 expression in CON, RNS, RNS + virus at 9 weeks (*n* = 6, *: vs. CON group, #: vs. RNS group, one‐way ANOVA with Bonferroni post hoc). (F–G) Hippocampus injection of virus vector LV‐Plppr4 (PRG‐1 OE) alleviated RNS‐induced TWL and MWT decrease while LV‐Plppr4‐RNAi (PRG‐1 SI) led to intensified pain (*n* = 7; repeated measures two‐way ANOVA with Tukey's post hoc multiple comparisons test, *: vs. RNS+ blank virus group at the same time point; arrow indicates virus vector injection). (H–J) PRG‐1 OE reversed the anxiety‐like behaviors in RNS rats by 9 weeks, such as more times entering the open arm and a greater percentage of time spent in the open arm in EPM experiment (*n* = 7; I: Kruskal–Wallis test with a Dunn's multiple comparisons test; J: One‐way ANOVA with Bonferroni post hoc; *: vs. RNS+ blank virus group at the same time point). (K–M) PRG‐1 OE relieved anxiety‐like behaviors in RNS rats by 9 weeks, including more times in the central area and a greater percentage of time spent in the central zone in OFT (*n* = 6; one‐way ANOVA with Bonferroni post hoc; *: vs. RNS+ blank virus group at the same time point). Data are presented as mean ± SEM with *p < 0.05. EPM, elevated plus maze; MWT, mechanical withdrawal threshold; OE, overexpression; OFT, open field test; RNS, repetitive noxious stimuli; SI, silencing; TWL, thermal withdrawal latency.

The expression of total PRG‐1 was transiently upregulated at postnatal Week 3, but decreased at Week 9 in the hippocampus of RNS rats (Figure 2A,B). To investigate the role of PRG‐1 in RNS‐induced pain, we bilaterally injected lentiviral vectors (LV‐Plppr4 for PRG‐1 overexpression or LV‐Plppr4‐RNAi for PRG‐1 silencing) into the area between CA1 and DG of the hippocampus of RNS rats at postnatal Week 3 (Figure 2C–E). PRG‐1 overexpression alleviates RNS‐induced mechanical allodynia and thermal hyperalgesia from Week 5 to Week 9, while PRG‐1 silencing exacerbated pain (Figure 2F,G), indicating the analgesic effect of PRG‐1.

Furthermore, PRG‐1 overexpression also relieves RNS‐induced anxiety‐like behaviors until Week 9, evidenced by increased open‐arm exploration in the EPM experiment (Figure 2H–J) and central zone activity in the OFT (Figure 2K–M). These results demonstrated that hippocampal PRG‐1 alleviates RNS‐induced pain hypersensitivity and anxiety‐like behaviors.

### 2.3 PRG‐1 Alleviates RNS Induced Pain Hypersensitivity via Neuronal Plasticity

**FIGURE 3:**
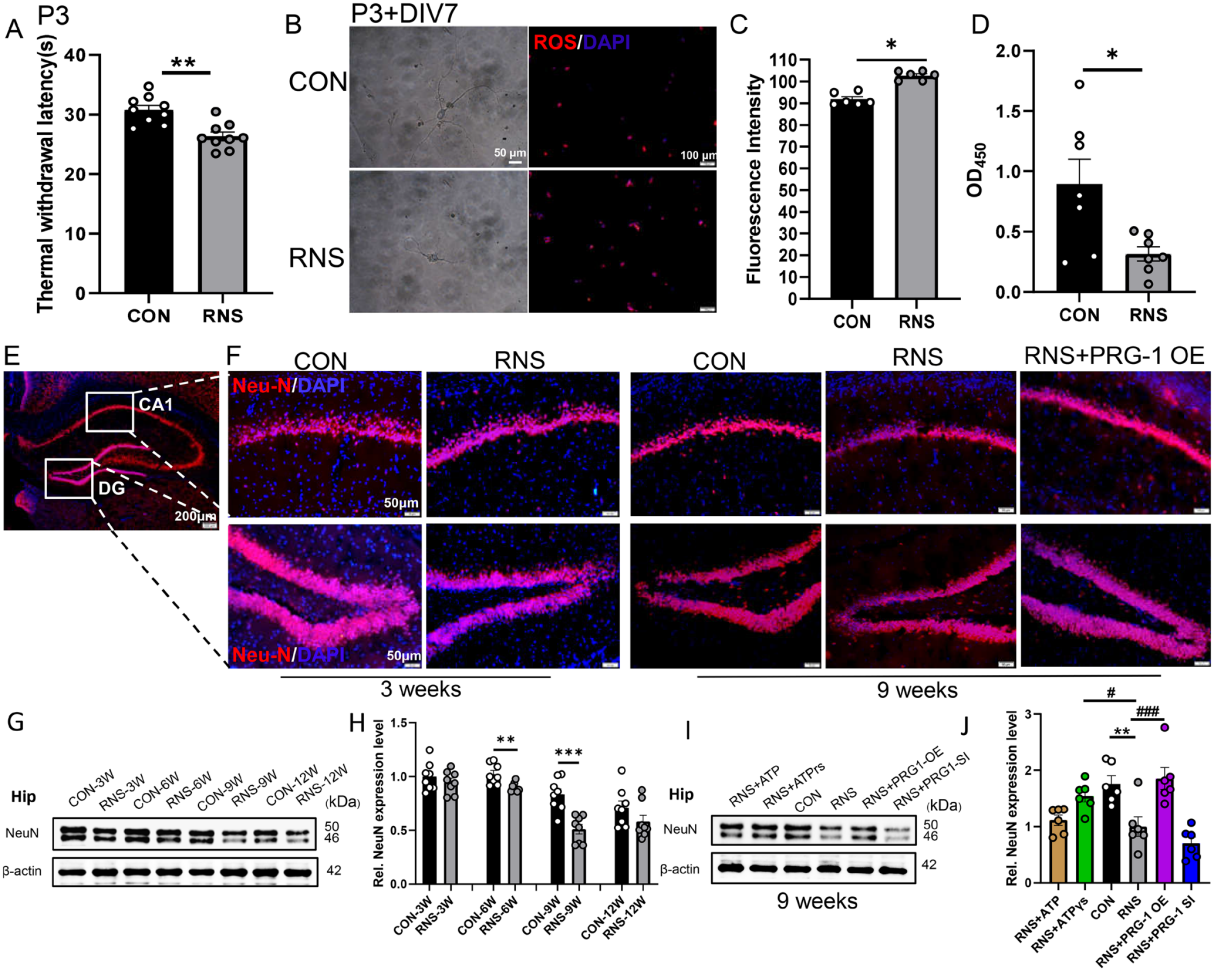
RNS rats display decreased neuronal activity in the hippocampus. (A) The TWL was significantly decreased in RNS rats at P3 (*n* = 8; two‐tailed unpaired student *t*‐test, *: vs. CON group at the same time point). (B, C) Damage evaluating in neurons by hydroethidine fluorescence staining with primary hippocampal neurons collected after neonatal RNS of P0–P3 and cultured in vitro for 7 days (DIV7) (*n* = 6, *: vs. CON group, two‐tailed unpaired student *t*‐test). (D) Apoptosis evaluating in neurons by CCK‐8 assay with primary hippocampal neurons culturing in DIV7 (*n* = 7, *: vs. CON group, two‐tailed unpaired student *t*‐test). (E) Overview of hippocampal section showing area of CA1 and DG (highlighted by white square). (F) Images of Neu‐N (red) and DAPI (blue) in the hippocampus by immunofluorescence at 3 and 9 weeks (scale bar = 50 μm). (G) WB bands and (H) quantitative analysis of NeuN expression in hippocampus in different periods (*n* = 8, *: vs. corresponding CON group, two‐tailed unpaired student *t*‐test). (I) WB bands and (J) quantitative analysis of NeuN expression in hippocampus after virus injection at 9 W (*n* = 6, *: vs. CON group, #: vs. RNS group, one‐way ANOVA with Bonferroni post hoc). Data are presented as mean ± SEM with n.s. *p* > 0.05; *p < 0.05; **p < 0.01; ***p < 0.001. OE, overexpression; RNS, repetitive noxious stimuli; SI, silencing.

Building on established roles of PRG‐1 in neuronal plasticity [16, 17], we elucidated its protective effects against RNS‐induced acute and long‐term neuronal damage. Primary hippocampal neurons were collected at P3 after neonatal RNS of P0–P3, which caused thermal hyperalgesia in rats (Figure 3A), and cultured in vitro for 7 days (DIV7) (Figure 3B). We evaluated the acute effects of RNS on neuronal apoptosis in neurons using CCK‐8 and damage by reactive oxygen species (ROS) assays, which are important markers of cellular oxidative damage due to physiological functions and environmental factors. RNS promoted ROS accumulation by hydroethidine fluorescence intensity (HFI) (Figure 3B,C) and revealed over 50% neuronal death by CCK‐8 results (Figure 3D) as acute effects in primary hippocampal neurons, implying RNS inducing hyperalgesia in rats through acute neuronal damage in neuronal developmental potential.

We also evaluated the long‐term neuronal damage of RNS by Neu‐N, a mature neuronal marker. The immunofluorescence staining revealed that NeuN expression was significantly decreased in the hippocampus CA1 and DG regions, which exhibit a causal role in controlling neuropathic pain‐related behaviors of RNS rats at 3 and 9 weeks (Figure 3E,F), and the total expression of NeuN was reduced in the hippocampus of RNS rats by western blots (Figure 3G,H), in line with reported results that pain resulted in pyramidal neuron atrophy and the number of neurons decreased in the hippocampus [23, 24]. PRG‐1 overexpression and ATPγS (enhanced PRG‐1/NSF interaction [17]) rescued NeuN loss in the hippocampus of RNS rats at 9 weeks (Figure 3F,I,J). Therefore, PRG‐1 may alleviate RNS‐induced pain hypersensitivity via preventing maladaptive neuronal plasticity.

### 2.4 PRG‐1 Relieves RNS Induced Pain Hypersensitivity via Synaptic Remodeling

**FIGURE 4:**
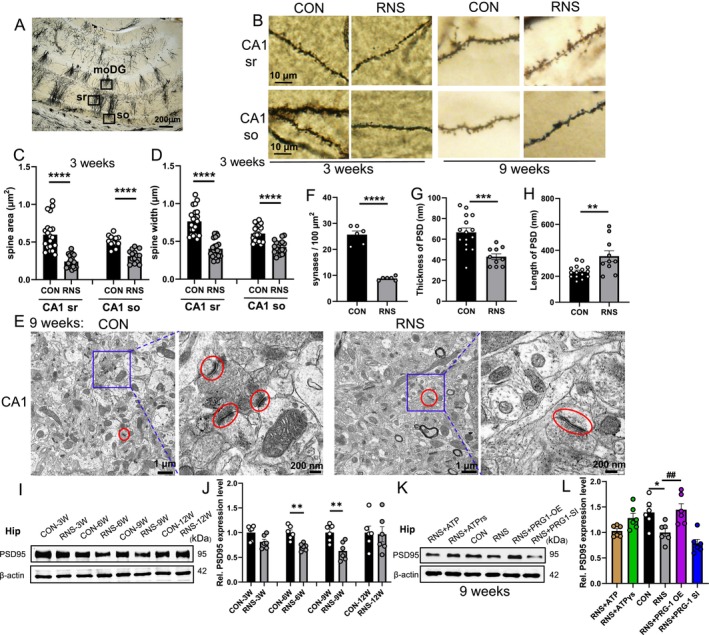
RNS rats display decreased spine area at 3 weeks in the hippocampus. (A) Hippocampus showing areas of spine assessment (highlighted by black squares): Stratum radiatum (sr, apical dendrites) and stratum oriens (so, basal dendrites) of the CA1 region. (B) Golgi‐Cox staining and (C, D) quantitative analysis showed that the spine area and spine width were decreased in the hippocampus of RNS rats at 3 W (C), *n* = 21 CON and 21 RNS in CA1 sr, 12 CON and 14 RNS at CA1 so; (D), *n* = 21 CON and 21 RNS in CA1 sr, 17 CON and 18 RNS at CA1 so; *n* represents analyzed dendritic segments; measurements for the spine length and width were averaged per dendritic segment; C left: Mann–Whitney‐*U* test; C right and D: Two‐tailed unpaired student *t*‐test, *p < 0.05, **p < 0.01, ***p < 0.001 compared to the CON group at the same time point. (E) Transmission electron microscopy and (F–H) quantitative analysis showed that the synapse number and thickness of PSD (highlighted by red circles) at the postsynaptic membrane were decreased in the hippocampus of RNS rats at 9 W (F, *n* = 6; G–H, *n* = 15 CON and 10 RNS; two‐tailed unpaired student *t*‐test, **p < 0.01, ***p < 0.001, ****p < 0.0001 compared to the CON group). (I) WB bands and (J) quantitative analysis of PSD 95 expression in the hippocampus in different periods (*n* = 6, *: vs. corresponding CON group, two‐tailed unpaired student *t*‐test). (K) WB bands and (L) quantitative analysis of PSD 95 expression in the hippocampus after virus injection at 9 W (*n* = 6, *: vs. CON group, #: vs. RNS group, one‐way ANOVA with Bonferroni post hoc). Data are presented as mean ± SEM with n.s. *p* > 0.05; *p < 0.05; **p < 0.01; ***p < 0.001; ****p < 0.0001. OE, overexpression; PSD, postsynaptic density; RNS, repetitive noxious stimuli; SI, silencing.

PRG‐1 is a critical regulator of synaptic plasticity [14]. Building on our prior observation of reduced hippocampal dendritic spine density following neonatal RNS [17], we systematically investigated RNS and PRG‐1 mediated alterations in synaptic architecture through morphometric analysis. Golgi‐Cox staining and quantitative analysis revealed time‐dependent morphological changes in hippocampal dendritic spines, including spine area and head width decreased in RNS rats at 3 W (Figure 4A–D) but not in 9 W. Furthermore, transmission electron microscopy uncovered distinct synaptic abnormalities at 9 weeks, including synaptic loss (synaptic density decreased by 66% in RNS rats) and postsynaptic density (PSD) remodeling (PSD thickness reduced by 35%, while PSD length increased by 51%) (Figure 4E–H). These structural anomalies indicate maladaptive synaptic plasticity driven by RNS.

The western blots revealed a significantly reduced expression of hippocampal PSD protein‐95 (PSD95), a scaffold protein derived from the origin of postsynaptic compact regions, in RNS rats at 6 and 9 weeks (Figure 4I,J). PRG‐1 overexpression fully restored PSD95 levels (Figure 4K,L), suggesting its therapeutic potential against RNS‐induced synaptic dysfunction.

### 2.5 AMPA Receptors Participate in the Analgesic Effect of PRG‐1 on RNS Induced Pain

**FIGURE 5:**
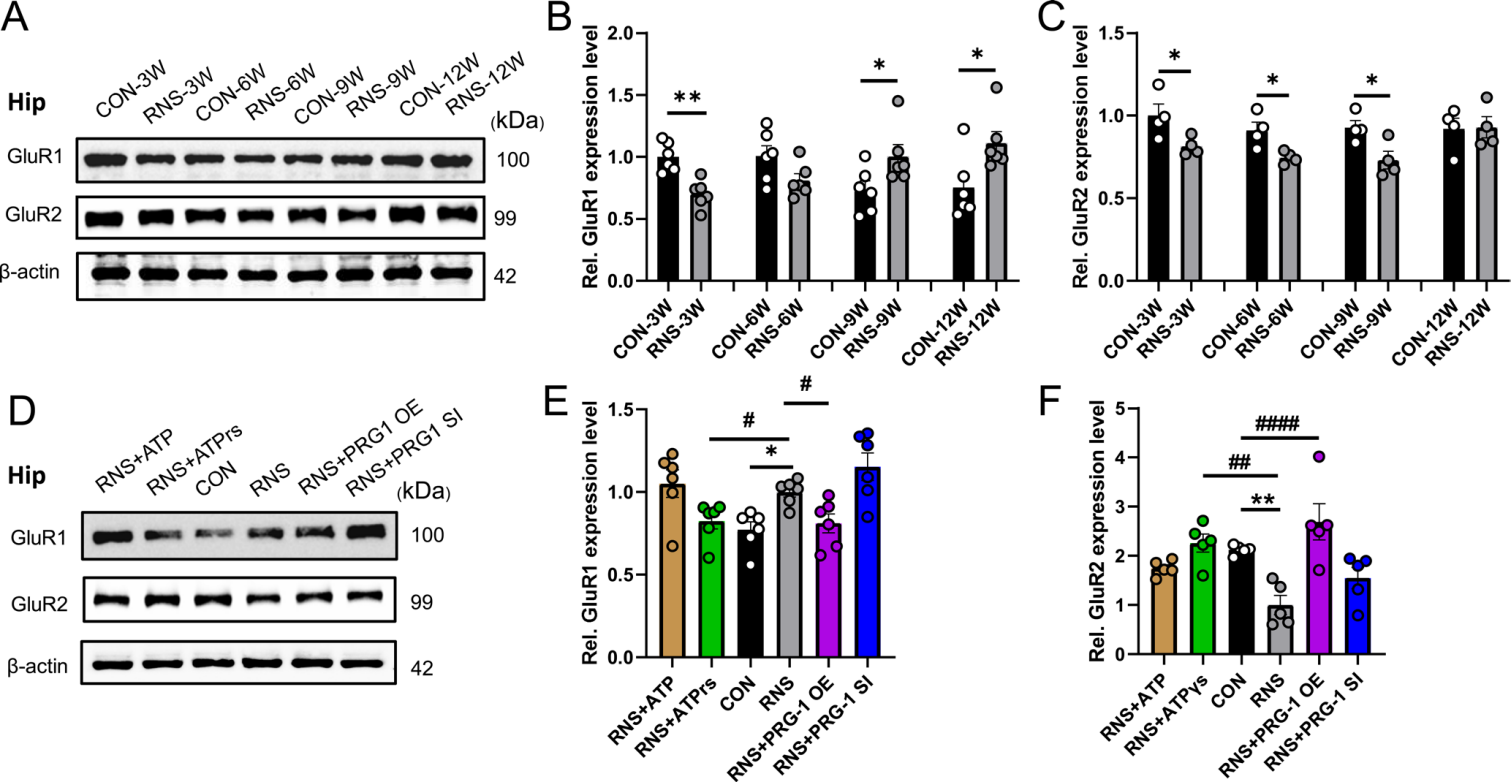
PRG‐1 relieves RNS induced pain in rats via AMPAR. (A) Western blot and (B, C) quantitative analysis of gray values showing GluR1 and GluR2 expression in different periods (*: vs. CON group, B: *N* = 6, 3–6 W: Mann–Whitney‐*U* test; 9–12 W: Two‐tailed unpaired student *t*‐test; C: *N* = 4, two‐tailed unpaired student *t*‐test). (D) Western blot and (E, F) quantitative analysis of gray values showing GluR1 and GluR2 expression in CON, RNS, RNS+ virus or drug group at 9 weeks (*: vs. CON group, #: vs. RNS group, E: *N* = 6, Kruskal–Wallis test with a Dunn's multiple comparisons test, F: *N* = 5, one‐way ANOVA with Bonferroni post hoc). Data are presented as mean ± SEM with *p < 0.05, **p < 0.01, ****p < 0.0001. OE, overexpression; RNS, repetitive noxious stimuli; SI, silencing.

PRG‐1 critically regulates glutamate release [15] and activity‐dependent trafficking of the α‐amino‐3‐hydroxy‐5‐methyl‐4‐isoxazole propionate receptors (AMPARs) [25]. Our prior work demonstrated that PRG‐1/NSF interaction relieves RNS‐induced pain in rats [17], while NSF also mediates synaptic sorting and transport of NMDARs and AMPARs [26]. A hallmark of silent synapses is the dominance of GluN2B‐containing NMDARs, which lack functional AMPARs. In the adult brain, AMPARs predominantly exist as heteromeric GluR1/GluR2 complexes, which confer Ca^2+^ impermeability (CI‐AMPARs) and mediate synaptic transmission and plasticity [27]. Hippocampal GluR2 expression was significantly reduced in RNS rats from 3 to 9 weeks, whereas GluR1 expression showed biphasic changes: decreased at 3 weeks but elevated at 9 and 12 weeks (Figure 5A–C). This temporal pattern indicates two distinct phases of synaptic remodeling: reduced GluR2 and GluR1 creates AMPAR‐deficient “silent synapses” in the early phase (3 weeks) and GluR1 upregulation drives silent‐to‐functional synapse conversion via GluR1 homomeric incorporation in the late phase (9 weeks). PRG‐1 overexpression and ATPγS treatment normalized GluR1/GluR2 stoichiometry at 9 weeks (Figure 5D–F), correlating with synapse remodeling. These findings reveal a PRG‐1‐dependent mechanism for AMPAR plasticity that prevents RNS‐induced maladaptive synapse accumulation.

### 2.6 PRG‐1 Ameliorates RNS Induced Pain in Rats via NMDARs

**FIGURE 6:**
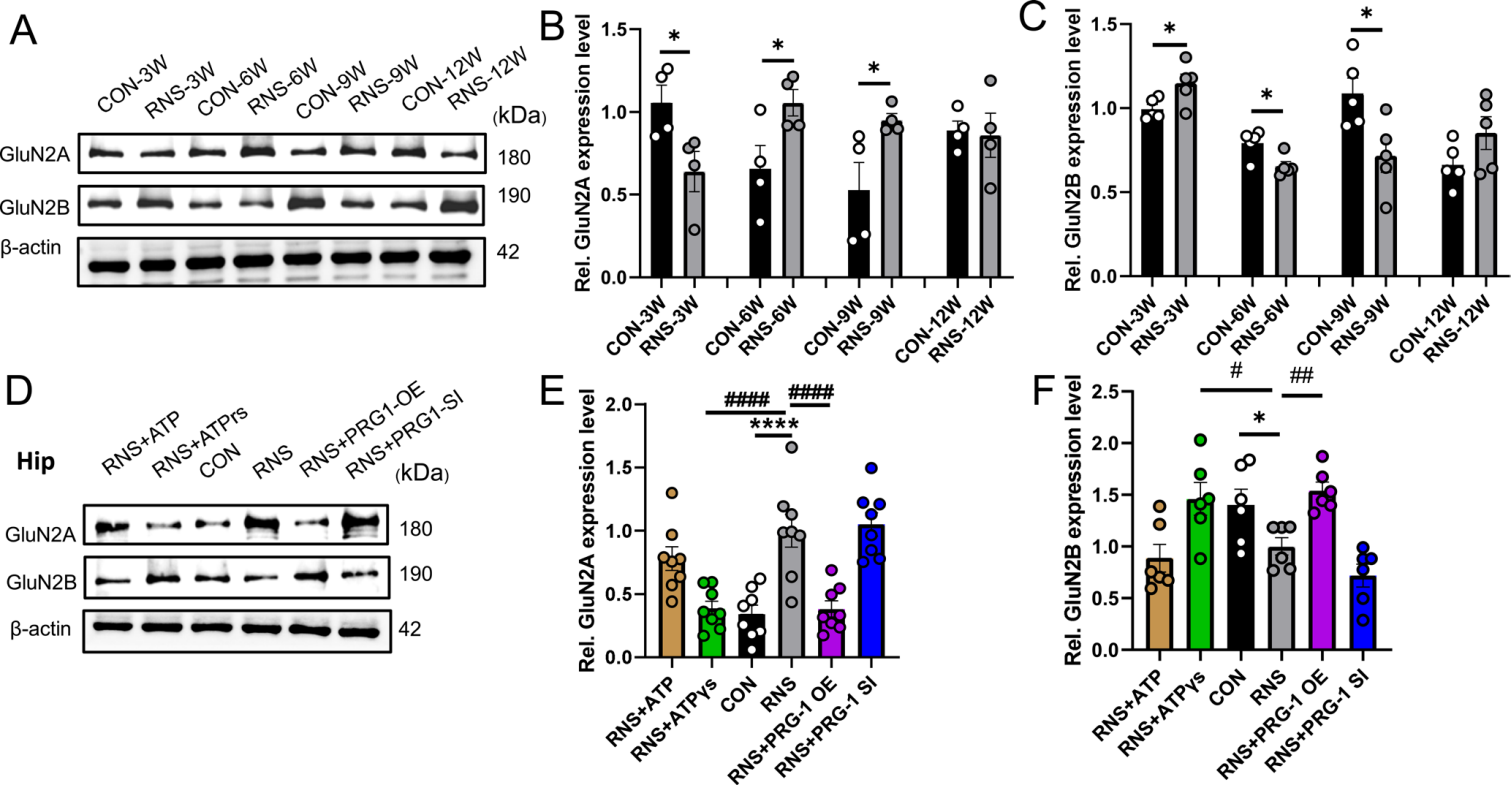
PRG‐1 relieves RNS induced pain in rats via NMDAR. (A) Western blot and (B, C) quantitative analysis of gray values showing GluN2A and GluN2B expression in different periods (B: *N* = 4, C: *N* = 5, *: vs. CON group, two‐tailed unpaired student *t*‐test). (D) Western blot and (E, F) quantitative analysis of gray values showing GluN2A and GluN2B expression in CON, RNS, RNS+ virus, or drug (ATPγS or ATP: 100 μM per day, P60–62) group at 9 weeks (E: *N* = 7, F: *N* = 6, *: vs. CON group, #: vs. RNS group, Kruskal–Wallis test with a Dunn's multiple comparisons test). Data are presented as mean ± SEM with *p < 0.05, **p < 0.01, ****p < 0.0001. OE, overexpression; RNS, repetitive noxious stimuli; SI, silencing.

*N*‐methyl‐D‐aspartate receptors (NMDARs) are central regulators of excitatory neurotransmission at synapses and are associated with severe neurological and psychiatric disorders [28]. The expression of the mature synaptic marker GluN2A was decreased at 3 W but increased at 6 W and 9 W, while the immature synaptic marker GluN2B was elevated at 3 W but reduced at 6 W and 9 W in RNS rats (Figure 6A–C). This biphasic shift resulted in a time‐dependent GluN2A/GluN2B imbalance in RNS rats: immature or silent synapses formed at an early phase (3 W) and partial synapse maturation at a late phase (6–9 W). PRG‐1 overexpression and ATPγS treatment may relieve RNS‐induced pain in rats via reversing alterations in the expression of NMDA receptors (Figure 6D–F), correlating with synapse reconstruction.

### 2.7 PRG‐1 Relieves Hyperalgesia in RNS Rats via Ca^2+^

**FIGURE 7:**
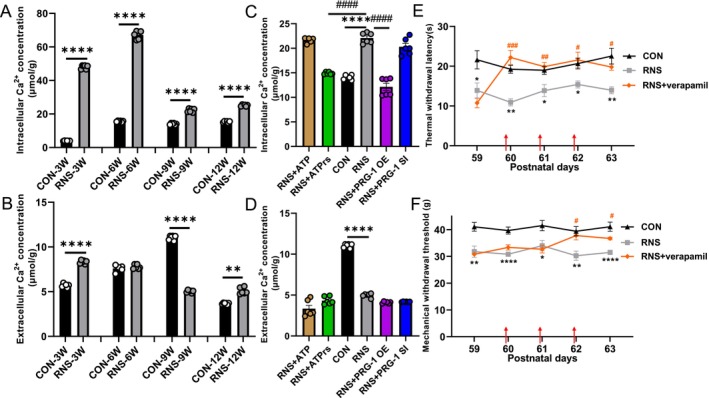
PRG‐1 relieves hyperalgesia in RNS rats via Ca^2+^. (A) ELISA shows that intracellular Ca^2+^ contents were increased in the hippocampus of RNS rats at 3, 6, 9, and 12 weeks compared with the age‐matched CON group (*n* = 6, two‐tailed unpaired student *t*‐test, *: vs. CON group). (B) ELISA shows that ATPγS or PRG‐1 overexpression induced intracellular Ca^2+^ content decreased in the hippocampus at 9 weeks in RNS rats (*n* = 6, one‐way ANOVA with Bonferroni post hoc; *: vs. CON group; #: vs. RNS group). (C) ELISA showing extracellular Ca^2+^ contents in CON and RNS groups at 3, 6, 9, and 12 weeks (*n* = 6, two‐tailed unpaired student *t*‐test, *: vs. CON group). (D) Virus or drug did not affect extracellular Ca^2+^ content in RNS rats. (E, F) Hippocampus injection of verapamil (100 μM per day, P60–62) alleviated RNS‐induced TWL and MWT decrease (*n* = 6, two‐way ANOVA with Tukey's post hoc multiple comparisons test, *: vs. CON group; #: vs. RNS group). Data are presented as mean ± SEM with *p < 0.05, **p < 0.01, ****p < 0.0001; RNS, repetitive noxious stimuli; OE, overexpression; SI, silencing.

After determining that PRG‐1 ameliorates RNS‐induced pain in rats via AMPAR and NMDAR, which may change the Ca^2+^ permeable situation, we quantified Ca^2+^ dynamics in the hippocampus. Enzyme‐linked immunosorbent assay (ELISA) analysis showed that intracellular Ca^2+^ contents were increased in the hippocampus of RNS rats across developmental stages (3–12 weeks) compared with the age‐matched CON group (Figure 7A). Furthermore, PRG‐1 overexpression and ATPγS treatment significantly attenuated intracellular Ca^2+^ overload at 9 weeks in RNS rats (Figure 7B), suggesting that PRG‐1 relieves RNS‐induced pain in rats via Ca^2+^. However, PRG‐1 expression intervention or relation drugs did not affect extracellular Ca^2+^ content in RNS rats (Figure 7C,D). Hippocampus bilateral injection of verapamil (100 μM per day, P60–62), a kind of calcium channel blocker, attenuated RNS‐induced mechanical allodynia and thermal hyperalgesia (Figure 7E,F), confirming that PRG‐1‐mediated Ca^2+^ regulation is critical for RNS‐induced pain relief.

### 2.8 PRG‐1 Relieves Hyperalgesia in RNS Rats via Modulation of Neuronal Firing Properties

**FIGURE 8:**
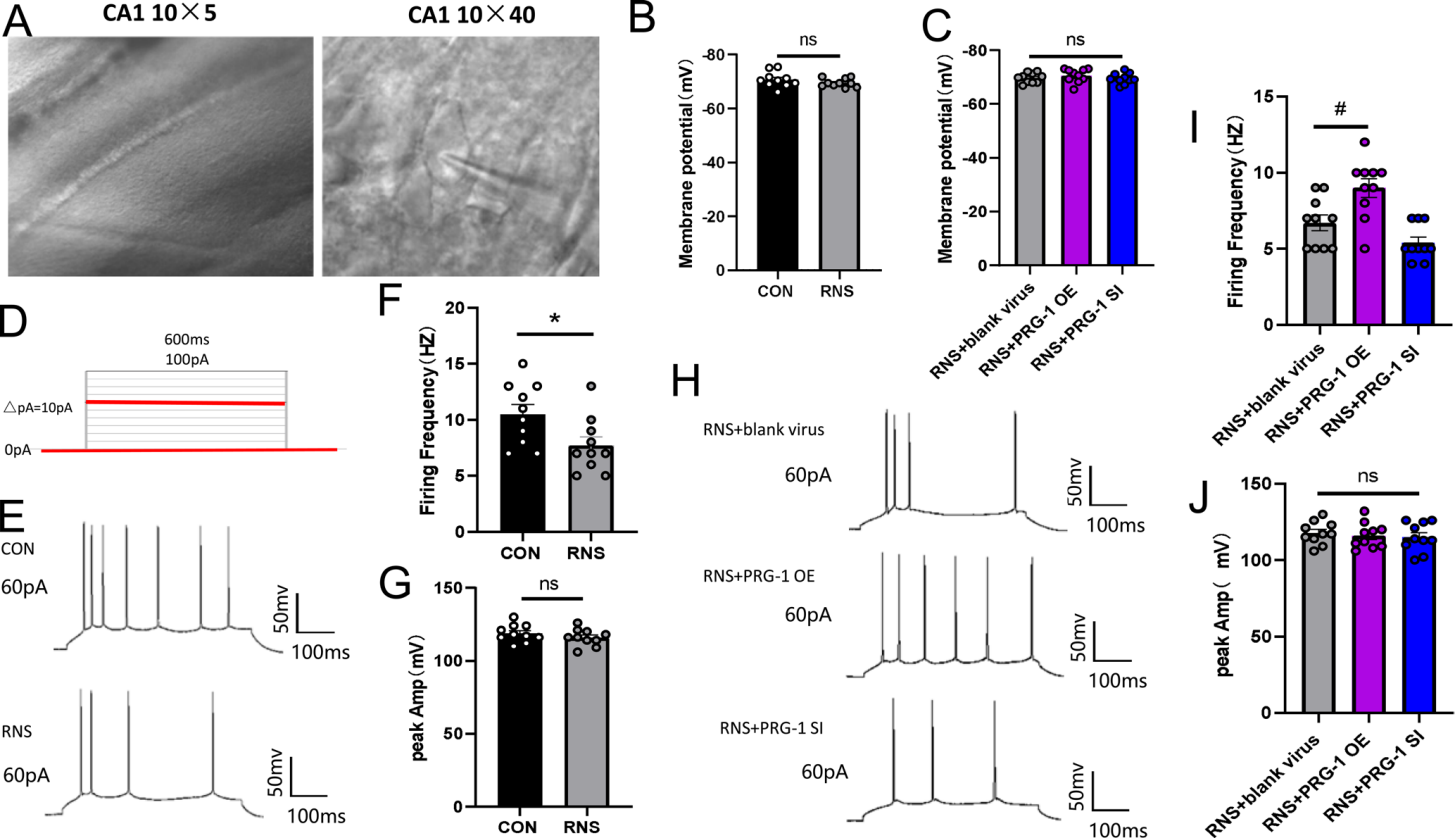
PRG‐1 relieves hyperalgesia in RNS rats by influencing neuronal firing properties. (A) distribution of neurons in the CA1 area and schematic diagram of hippocampal pyramidal neurons (magnification: 10 × 5 in left and 10 × 40 in right). (B) Membrane potential of pyramidal neurons in the CA1 area of CON and RNS rats (*n* = 10, *: vs. CON group, two‐tailed unpaired student *t*‐test, *p* > 0.05). (C) Membrane potential of pyramidal neurons in the CA1 area of PRG‐1 expression intervention rats (*n* = 10; one‐way ANOVA with Bonferroni post hoc, *p* > 0.05). (D) Plot of 0–100 pA current stimulation of pyramidal neurons in the CA1 area. (E–G) Diagram of firing (E), firing frequency statistics (F) and firing amplitude (G) in the CA1 area of CON and RNS rats under 60 pA stimulation (*n* = 10, *: vs. CON group, two‐tailed unpaired student t‐test). (H–J) Diagram of action potential (H), firing frequency (I) and firing amplitude statistics (J) in the CA1 area of PRG‐1 expression intervention rats under 60 pA stimulation (*n* = 10, #: vs. RNS+ blank virus group, I: Kruskal–Wallis test with a Dunn's multiple comparisons test, J: One‐way ANOVA with Bonferroni post hoc). Data are presented as mean ± SEM with ns *p* > 0.05, *p < 0.05. OE, overexpression; RNS, repetitive noxious stimuli; SI, silencing.

Neonatal acupuncture has been shown to enhance dorsal horn neuronal hyperexcitability [29]. The whole‐cell patch clamp of pyramidal neurons in the hippocampal CA1 region was recorded at 3–4 W of CON and RNS rats or 5–6 W of PRG‐1 intervention groups, with virus injected bilaterally into the hippocampus at 3 W (Figure 8A). No significant differences in resting membrane potential were observed among CON, RNS, and PRG‐1 intervention groups (Figure 8B,C). However, under 60 pA current stimulation, the firing frequency of action potential decreased in the CA1 area of the RNS group (Figure 8D–F), illustrating reduced responses to external stimuli in RNS rats at an early stage, while no significant difference in firing amplitude was observed (Figure 8G). PRG‐1 overexpression reverses RNS‐induced reductions in action potential firing frequency in hippocampal CA1 neurons (Figure 8H–J), indicating that PRG‐1 relieves hyperalgesia in RNS rats via modulation of neuronal firing properties.

### 2.9 PRG‐1 Reverses Hyperalgesia in RNS Rats Through Synaptic Transmission

**FIGURE 9:**
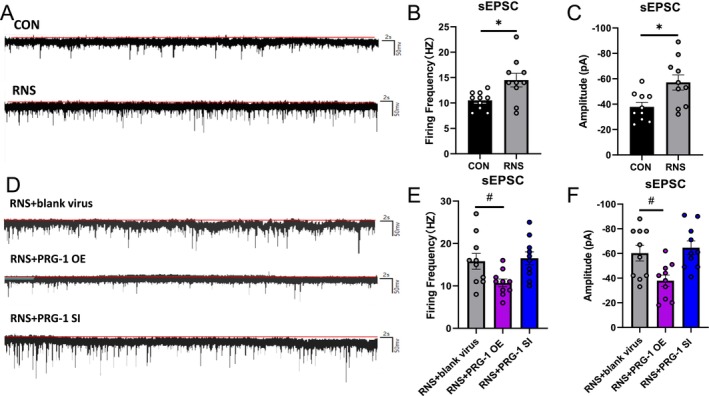
PRG‐1 relieves hyperalgesia in RNS rats through synaptic transmission. (A–C) Schematic diagram of sEPSC (A), frequency statistics (B) and amplitude statistics (C) of pyramidal neurons in the CA1 area of CON and RNS rats (*n* = 10, *: vs. CON group, two‐tailed unpaired student *t*‐test). (D, E) Schematic diagram of sEPSC (D), frequency statistics (E) and amplitude statistics (F) of pyramidal neurons in the CA1 area of PRG‐1 expression intervention rats (*n* = 10, #: vs. RNS+ blank virus group, E: one‐way ANOVA with Bonferroni post hoc). Data are presented as mean ± SEM with *p < 0.05. OE, overexpression; RNS, repetitive noxious stimuli; sEPSC, spontaneous excitatory postsynaptic potential; SI, silencing.

Synaptic plasticity encompasses both structural and functional plasticity, quantifiable through electrophysiological properties. Electrophysiological properties of the hippocampal CA1 synapse showed an increase in spontaneous excitatory postsynaptic potential (sEPSC) firing frequency and peak amplitude in RNS rats at 3–4 W (Figure 9A–C), indicating RNS mediates abnormal synaptic transmission, including hyperactive presynaptic neurotransmitter release and postsynaptic response sensitization. PRG‐1 overexpression attenuated aberrant excitatory synaptic transmission (Figure 9D–F). These findings demonstrate that PRG‐1 alleviates hyperalgesia by restoring synaptic transmission fidelity and preventing maladaptive plasticity in RNS rats.

## 3 Discussion

Infant analgesia is often overlooked, which may lead to dysplasia of the immature nervous system and short‐ and long‐term structural and functional maladaptive neuronal remodeling of neonates in the NICU [1, 7, 8], manifesting as altered pain sensitivity, emotional, and psychosocial sequelae later in life [30, 31, 32, 33]. However, the mechanisms underlying early painful experience‐induced pain sensitivity and emotional problems later in life remain elusive. Here, we used a neonatal RNS rat model to elucidate the temporal dynamic impact of neonatal RNS on later life.

RNS caused significant decreases in TWL and MWT values and anxiety‐like behaviors from Week 1 to Week 10 but no dominance difference at Week 12, suggesting that neonatal RNS induces persistent thermal hyperalgesia and mechanical allodynia but not permanently in rats. RNS‐IN (incisions at Week 12) rats exhibited heightened mechanical allodynia and thermal hyperalgesia from Day 1 to Day 14, which means RNS primed sensitization of postoperative pain in adulthood by stealth pain memory at Week 12 rather than dominance pain or anxiety, aligning with the fact that surgery in the neonatal period leads to sensitization of pain, increased dosage of analgesics, and increased incidence and intensity of recurrent pain after surgery in adulthood [2]. Though quite compelling, the relationships between body weight, depression, and anxiety are far from concrete or universal [34, 35, 36]. In our study, we do not find an association between body weight and RNS‐induced anxiety. The reason may be that the weight status is affected by many factors.

To date, studies on PRGs family have mainly focused on neuropsychiatric diseases and nerve injuries including epilepsy [15, 37, 38, 39], schizophrenia [40, 41, 42], memory disorders [14], nerve trauma [43, 44], sensory discrimination deficit [45, 46] and fasting‐induced hyperphagia [47]. We recently reported that PRG‐1 relieves bone cancer pain and depressive‐like behaviors in rats by dendritic spine in hippocampus [16]. PRG‐1 overexpression alleviates RNS‐induced mechanical allodynia, thermal hyperalgesia, and anxiety‐like behaviors, while PRG‐1 silencing caused exacerbated pain (Figure 2F,G), indicating the analgesic and anti‐anxiety effect of PRG‐1.

Interestingly, acute and long‐term effects of neonatal incision are different. Neonatal hind paw incision elevates Iba1 positive cells in the spinal dorsal horn and produces acute hyperalgesia in neonatal rats [48], while no impact of Iba1 in the dorsal horn was observed in adults [49] and long‐term global hypoalgesia is produced [2, 50]. In our study, acute and long‐term effects of neonatal RNS are also different. PRG‐1 expression is down‐regulated by RNS because of the similar phenotype with PRG‐1 knockout mice [14]. PRG‐1 expression is then temporarily upregulated following RNS due to compensatory protection in response to RNS‐induced pain. The combination of these two factors leads to substantial up‐regulation of PRG‐1 expression at week 3 in line with reported results [12, 51]. Over the subsequent few weeks, this self‐protective effect weakens, leading to no significant alterations at Week 6 and the decline at Week 9 of PRG‐1 in the hippocampus. However, the acute compensatory increase of PRG‐1 could not completely offset the neurodevelopmental disorders and nerve damage caused by RNS, which still manifest as hyperalgesia and anxiety‐like behavior.

Another interesting finding is the incomplete match of PRG‐1 expression levels, neuronal markers, and their functional consequences. This may be partially due to the stage‐specific coordination that is affected by a biphasic response to neonatal RNS. Initially, RNS‐induced pain causes a sharp decline in hippocampal spine size and altered synaptic transmission efficacy. In the second phase, higher PRG‐1 concentrations caused by the compensatory response at 3 weeks are an attempt to mitigate the dendritic spine damage and relieve the pain and anxiety‐like behavior. In the absence of intervention, however, this second compensatory phase is weak under natural conditions. Interventions such as ectopic overexpression of PRG‐1 or pharmacologic ATPγS administration to enhance PRG‐1/NSF can normalize dendritic spine and mitigate the pain and anxiety‐like behavior induced by neonatal RNS.

Typical silent synapses contain GluN2B receptors and lack functional AMPARs, while transport and insertion of AMPAR subunits and GluN2A receptors and overexpression of PSD‐95 protein can convert silent synapses into functional synapses [52]. Neuropathic pain due to chronic constriction injury (CCI) induces silent synapse formation in the short term (7 d), characterized by increased immature dendritic spine density and NMDA receptors containing synapses in anterior cingulate cortex (ACC) neurons, followed by decreased silent synapses after a long time (30 d) [19]. Similarly, RNS triggers transient silent or immature synapse generation in the short term (3 W), with an increase in GluN2B and a decrease in AMPARs (both GluR1 and GluR2), which may cause the formation of hyperalgesia. After a long time (9 W), silent or immature synapses transit to functional synapses evidenced by increased mature synapses containing GluN2A (an increase in GluN2A and decrease in GluN2B), causing intracellular Ca^2+^ overload and AMPA receptors in hippocampal neurons. However, AMPARs consist of heteromeric GluR1/GluR2 subunits (Ca^2+^ impermeable) under physiological conditions [27] were shifted to GluR1 subunits (Ca^2+^ permeable) by RNS (an increase in GluR1 and decrease in GluR2), which also causes intracellular Ca^2+^ overload and dramatically alters synaptic function. These changes lead to the maintenance of persistent hyperalgesia and formation of pain memory.

Action potential is a membrane potential across a cellular membrane when stimulated. In our experiment, action potential firing frequency under current stimulation of 60 pA of RNS rat neurons was significantly lower than that of the CON group, indicating more silent synapses and reduced responses to external stimuli in RNS rats at the early stage. RPG‐1 overexpression could turn silent synapses into functional synapses, resulting in increased firing frequency and enhanced response to stimuli. Furthermore, we found an increase in the peak amplitude and firing frequency of sEPSC in RNS rats, implying RNS mediate abnormal synaptic transmission and overexcitability, in line with the phenotype of PRG‐1 knockout mice [14]. PRG‐1 abolishes the effects on the firing frequency of action potential and attenuates aberrant excitatory synaptic transmission, protecting synaptic plasticity.

**FIGURE 10:**
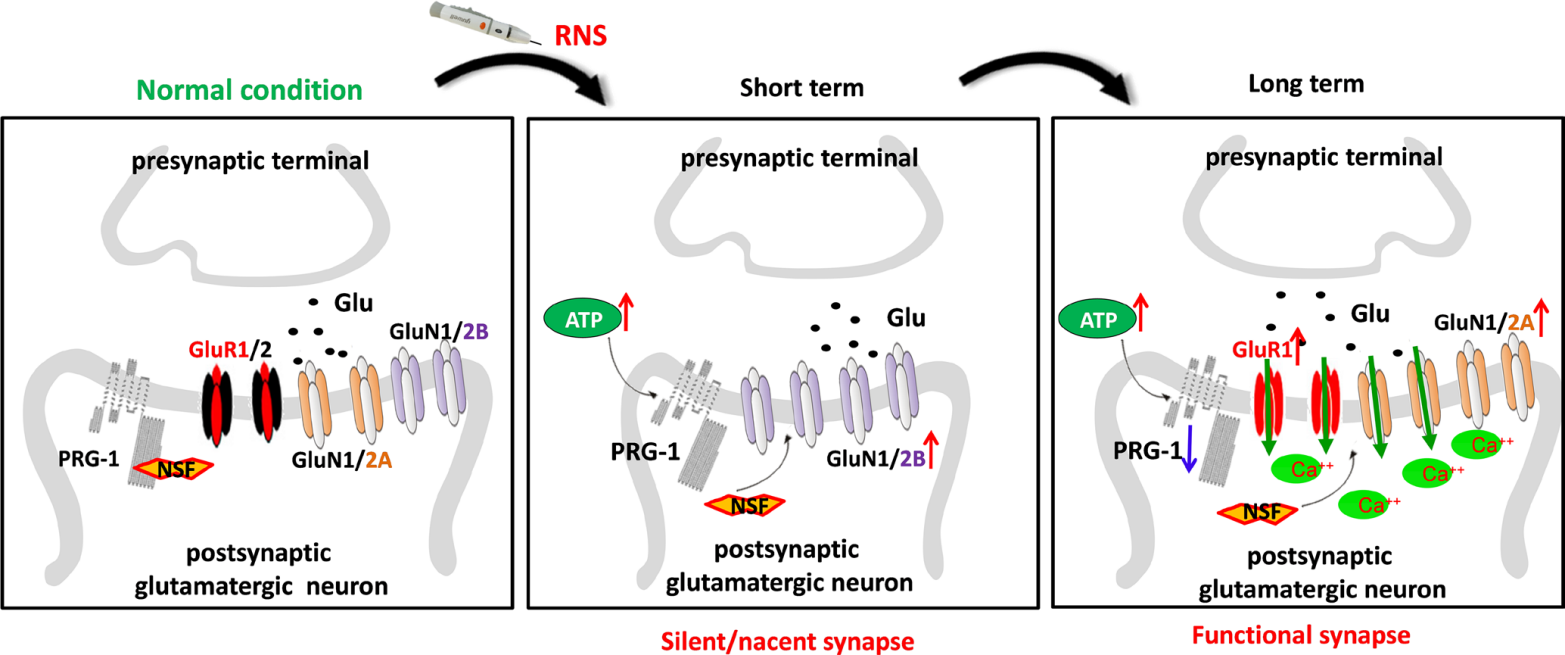
Schematic for the proposed mechanisms of PRG‐1 modulating RNS‐induced hyperalgesia and anxiety via synapse remodeling at different stages. Left: PRG‐1/NSF interactions maintain PRG‐1 function and neurotransmitter receptor stability under normal conditions. Middle: In the early stage of RNS, the release of extracellular ATP increases, with the inhibition of PRG‐1/NSF interaction, which causes the decrease of synaptic activity and area, implying the transformation into silent or immature synapse, causing the formation of hyperalgesia. Although the expression of PRG‐1 increases in the short term, it is still difficult to reverse the overall trend. Right: In the late stage of RNS, RNS stimulation leads to a decrease in PRG‐1 expression at later stages, and the extracellular ATP release continuously inhibits PRG‐1/NSF interaction; then it causes an altered AMPA and NMDA receptor composition at the postsynaptic membrane in the intracellular. This pathway causes a synapse transition to an abnormal functional synapse, leading to the maintenance of persistent hyperalgesia.

In line with these findings, our results demonstrated that in the early stage of RNS, the release of extracellular ATP increases, with the inhibition of PRG‐1/NSF interaction, which causes the decrease of synaptic activity and transforms into a silent or immature synapse (containing GluN2B and lack of AMPAR, decreased spine area and head width, reduced responses to external stimuli, aberrant excitatory synaptic transmission), causing the formation of hyperalgesia. Although the expression of PRG‐1 increases compensatorily in the short term, it is still difficult to reverse the overall trend. In the late stage, RNS stimulation leads to a decrease in PRG‐1 expression at later stages and the extracellular ATP release continuously inhibits the PRG‐1/NSF interaction, then causing an altered AMPAR (Ca^2+^ impermeable GluR1/GluR2 converted to Ca^2+^ permeable GluR1) and NMDAR (GluN2B converted to GluN2A) composition at the postsynaptic membrane and intracellular Ca^2+^ overload. This pathway causes a synapse transition to an abnormal functional synapse (decreased synapse number and thickness of PSD, increased length of PSD), leading to the maintenance of persistent hyperalgesia. PRG‐1 may relieve RNS‐induced pain in rats via stage‐specific reversing of AMPA and NMDA receptor balance, attenuating intracellular Ca^2+^ overload and remodeling the synapse structure and function (Figure 10).

In conclusion, here we found that PRG‐1 mediated stage‐specific synaptic remodeling contributes to the RNS induced pain via dynamically trafficking of AMPAR and NMDAR. The role of the PRG‐1 pathway in RNS induced pain depends on stage‐specific effects on GluR1/2 and GluN2A/B subunits and synapse function. PRG‐1 provides a pharmacological approach and strategy to afford RNS‐induced hyperalgesia, anxiety, and pain‐perception memory.

### 3.1 Limitations of the Study

In the present study, the differential roles of PRG‐1 in RNS mediating hyperalgesia and anxiety in different brain regions, such as PFC and ACC, need further investigation. Furthermore, the role of AMPAR and NMDAR in RNS reduced pain can be elucidated.

## 4 STAR Methods

### 4.1 Resource Availability

#### 4.1.1 Lead Contact

Further information and requests for resources and reagents should be directed to and will be fulfilled by the lead contact, Xingfeng Liu (xingfengliu@zmu.edu.cn).

#### 4.1.2 Materials Availability

Virus generated in this study has been deposited to Genechem, LV‐Plppr4, ID: LV‐Plppr4(47472‐1); Plppr4‐RNAi, ID: Plppr4‐RNAi (85438‐1).

This study did not generate new unique reagents.

#### 4.1.3 Data and Code Availability

- The data reported in this paper will be shared by the lead contact upon request.
- This paper does not report original code.
- Any additional information required to reanalyze the data reported in this paper is available from the lead contact upon request.

### 4.2 Experimental Model and Subject Details

#### 4.2.1 Animals

Pathogen‐free Sprague–Dawley (SD) adult rats were purchased from the Tianqin Biotechnology Co. Ltd. (license number: SCXK (Xiang) 2019‐0014; Changsha, China) and neonatal rats were obtained by pairing via normal breeding. Rats were housed in a temperature (23°C ± 2°C) and humidity (55% ± 5%)‐controlled environment, with a 12 h light/dark cycle (8:00 a.m.–8:00 p.m.) and ad libitum access to food and water. All experimental procedures were in accordance with the guidelines of the Ethical Committee of the International Association for the Study of Pain (IASP) [53] and ethically approved by the Animal Care Ethics Committee of Zunyi Medical University (zunyilunshen [2020] 2‐098). All viable efforts were made to minimize the use of animals and reduce their suffering from experimental procedures.

**DIAGRAM 1:**
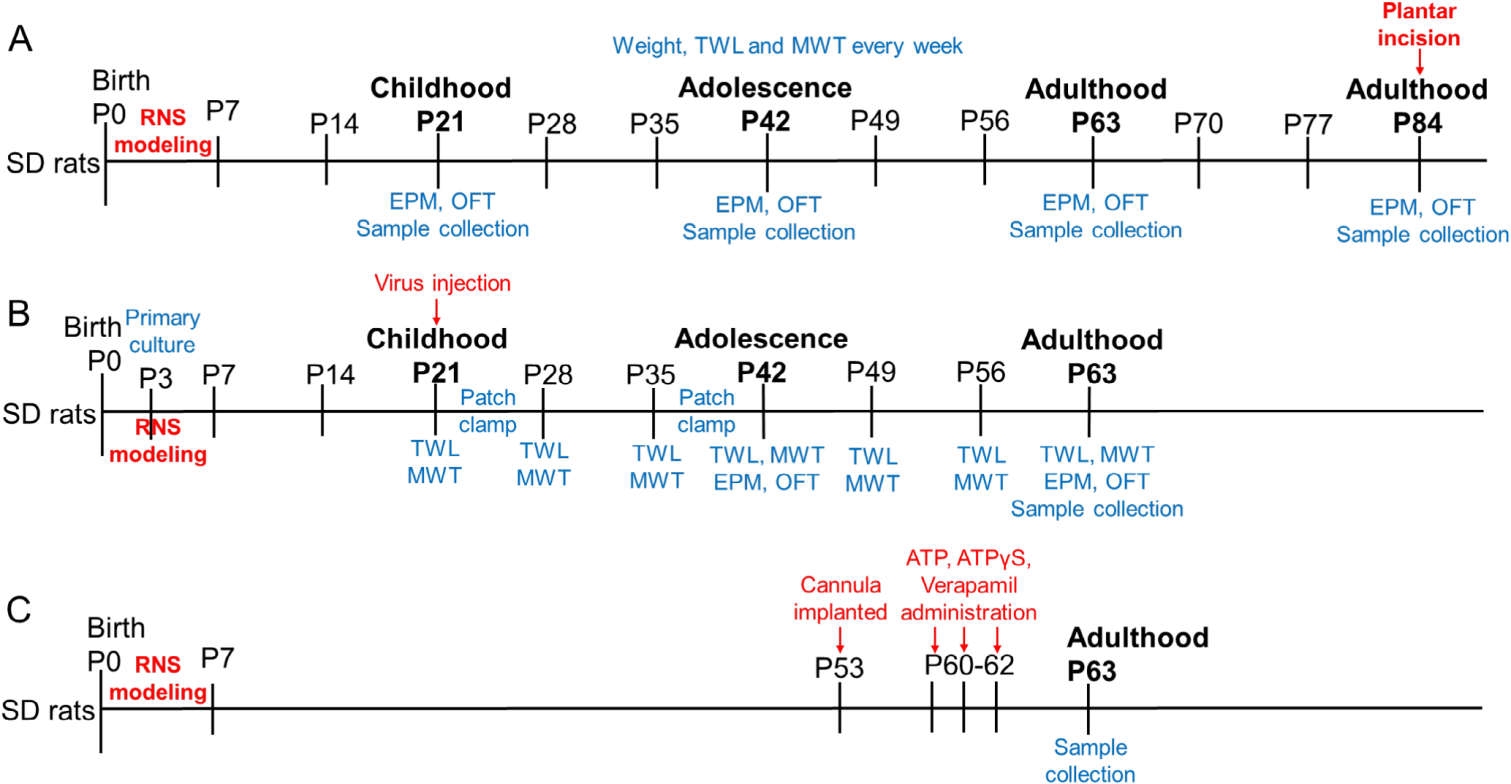
Schematic timeline and strategy of experimental design to elucidate effects of PRG‐1 on RNS‐induced pain. (A) Blue words indicate the time of the behavior test and sample collection. (B) Red arrow indicates the time of virus injection. (C) Red arrows indicate the time of drug administration.

Rat pups (both male and female) were randomly numbered and then allocated to experimental groups according to a computer‐generated randomization code. In our study, no sex differences were observed in RNS‐induced pain and associated internalizing behaviors, aligning with findings from some clinical studies [5, 54]. Consequently, sex was not stratified or adjusted as an independent variable in subsequent analyses. The overall experimental design is shown in the schematic diagram (Diagram 1). The grouping and number of rats are shown in Table S1. Cutaneous sensitivity to thermal and mechanical stimulation was measured every week after birth until 12 weeks of age (P7, 14, 21, 28, 35, 42, 49, 56, 63, 70, 77, and 84). The elevated plus‐maze test (EPM) and open‐field test (OF) were examined every 3 weeks after birth. RNS rats without pain were not included in the analysis, and the number of each group that failed to demonstrate impairments was substituted by additional RNS rats. The hippocampus was harvested at the appropriate time point for further analyses. The individual doing behavioral testing of the rats was blinded to the treatment groups of animals.

#### 4.2.2 Animal Model of Neonatal RNS

The neonatal RNS rat model was established as described [55] with some modifications. A 28G blood glucose needle was rapidly pricked into the middle plantar of rats' four feet in turn every 6 h at 0:00, 6:00, 12:00, and 18:00 from the day of birth (P0) to P7, and the control group received tactile stimulation with a cotton‐tipped swab at the same place and time interval. The pups were put back in the original cage after sufficient hemostasis by cotton swab pressing and disinfection with iodine before and after the prick to prevent infection and minimize the mother–child separation time. All rats were reared to P22, weaned, and housed in sex‐matched cages from P23.

#### 4.2.3 Animal Model of Plantar Incision

After anesthesia with 2%–3% isoflurane (Sigma‐Aldrich, USA), a plantar incision was performed in the right hind paw of adult rats (P84). A small incision extending from the mid‐point of the heel to the first footpad was made through the skin and fascia, and the underlying muscle was also elevated and incised. The length of the incision was approximately 10 mm in the adult rat, and the skin was immediately closed with sutures [2]. To mimic the neonatal RNS‐induced exaggeration of adult incisional pain, the experimental groups contain: (i) CON: control adult rats; (ii) RNS: adult rats having only RNS in the neonatal period; (iii) IN: rats having only adult incision; and (iv) RNS‐IN: rats having neonatal RNS and adult incision.

#### 4.2.4 Overexpression or Knockdown of PRG‐1

As we previously used [17], the sequence coding PRG‐1 (Plppr4, GeneBank number NM_001001508) was amplified from plasmid and cloned into pGV358 lentivirus vector. Lentivirus‐PRG‐1 (LV‐Plppr4) was generated to overexpress PRG‐1. Recombinant lentivirus‐short hairpin PRG‐1 (LV‐Plppr4‐RNAi) was constructed to knock down PRG‐1. Short hairpin RNA was generated using sense small interfering RNA sequence targeting PRG‐1. Lentivirus without PRG‐1 insertion was used as the control. Recombinant lentivirus was constructed by Genechem (China).

#### 4.2.5 Isolation, Culture and Analysis of Primary Hippocampal Neurons

Primary hippocampal neurons from postnatal Day 3 (P3) of CON and RNS rats were prepared as previously described [14]. Briefly, after hippocampus dissection and trypsin digestion, neurons triturated using fire polished glass pipets were seeded on poly‐l‐lysine (Solarbio) coated plates in Minimum Essential Medium (Gibco) supplemented with 10% fetal bovine serum, 100 U/mL penicillin, and 100 μg/mL streptomycin (Gibco) and kept at 37°C/5% CO_2_. After 4–6 h, neurons were washed once with pre‐warmed PBS and incubated with Neurobasal Medium (Gibco) supplemented with 2% B‐27, 100 units/mL penicillin, 100 μg/mL streptomycin, and 0.5 mM glutamine (Gibco). For analyses, neurons were analyzed by CCK‐8 assay (Solarbio, China) or SOD assay (vigorous, China) at days in vitro (DIV) 7.

### 4.3 Method Details

#### 4.3.1 Implantation of Hippocampal Cannula and Microinjection Procedures

After anesthesia with pentobarbital sodium (50 mg/kg body weight, intraperitoneal, i.p.), rats at 3 weeks of age were placed on a stereotaxic frame (RWD Life Science, China). The skull was exposed and the lentivirus vector LV‐Plppr4 or LV‐Plppr4‐RNAi (Genechem, Shanghai) was slowly injected (0.5 μL unilateral, 200 nL/min) bilaterally into the hippocampus area (AP = −3.10 mm, ML = ±1.7 mm, and DV = −2.5 mm), according to the rat brain atlas [56] with appropriate modification, via microsyringe pump (RWD Life Science, China). The pipette remained in place for 10 min to ensure complete diffusion of the virus and was then slowly removed [57].

For drug, including ATP (Sigma, Germany), ATPγS (Sigma, Germany) and verapamil (MCE, China) treatment experiments, a guide cannula (0.48 mm outside diameter [O.D.]; 0.34 mm inner diameter [I.D.]) was implanted into the bilateral hippocampus (AP = −3.72 mm, ML = ±2.0 mm, and DV = −3.0 mm), according to the rat brain atlas [56], and fixed to the skull of rats on P53. Rats were inserted with an injection cannula (0.3 mm O.D.; 0.5 mm longer than the guide cannula) into the guide cannula. A total volume of 0.5 μL drug solution was slowly injected once per day between P60–P62. Microinjection sites were checked by histological examination [58].

#### 4.3.2 Behavioral Assessment

##### 4.3.2.1 Radiant Heat Test

Thermal hyperalgesia was tested in rats as reported [59] previously to evaluate thermal withdrawal latency (TWL). Each rat was placed and adapted in an individual Plexiglass cube for 30 min; then 52°C ± 0.2°C radiant heat (50 W, 8 V bulb) was applied to the plantar surface of the hind paw with a plantar radiant heat instrument (Model 390; IITC Life Science Instruments, USA). Latency period was recorded until the removal of the paw. The cut‐off value was set to 60 s. Each hind paw was measured five times at 5‐min intervals [60].

##### 4.3.2.2 The Electronic von Frey Meter Test

Mechanical allodynia of rat hind paw was used to evaluate mechanical withdrawal threshold (MWT) with the electronic von Frey anesthesiometer (IITC Life Science Instruments, USA) as described [61]. Each rat was placed and adapted in an individual plastic chamber for 30 min. The polypropylene tip was applied to the plantar surface of the hind paw with a gradual increase in pressure until the removal of the paw. The threshold pressure was recorded. Each hind paw was measured five times at 5‐min intervals.

##### 4.3.2.3 Elevated Plus‐Maze Test (EPM)

The elevated plus‐maze (RWD life Science) consisted of two vertically crossed open arms (50 cm × 10 cm), two closed arms (50 cm × 10 cm × 40 cm) and a central area (10 cm × 10 cm). After 30 min adaptation, the rat was placed in the central area with its head facing the open arms and allowed to ad libitum explore for 10 min in the maze. The path, number of entries into the open arms, and the time in open arms of each rat were automatically recorded by a camera and analyzed with a computerized video Smart 3.0 system (Panlab, Spain).

##### 4.3.2.4 Open‐Field Test (OF)

Rats were placed in an empty chamber box with clear sidewalls (1 m × 1 m × 20 cm, RWD life Science) and allowed to freely explore for 10 min. Their locomotor activity was recorded by camera and analyzed with a computerized video Smart 3.0 system (Panlab, Spain).

##### 4.3.2.5 Perfusion and Immunofluorescence

Rats were deeply anesthetized with pentobarbital sodium (50 mg/kg body weight, i.p.) before the perfusion of normal saline (NS) through the left ventricle followed by 4% PFA (paraformaldehyde). Brains were dissected, post‐fixed in 4% PFA for 4–6 h at 4°C, and cryoprotected in 30% sucrose at 4°C until they sank. The brains were then coronally sliced at a thickness of 30 μm on a cryostat (Leica CM 1950, Germany).

For immunofluorescence, the sections were blocked and permeabilized, and then incubated with rabbit anti‐rat NeuN antibody (1:300; CST). Sections were incubated in CY3‐conjugated donkey anti‐rabbit antibody conjugated with CY3 (1:1000; Abcam). The captured images were observed under a fluorescence microscope (Olympus DP80, Japan).

##### 4.3.2.6 Western Blot (WB)

The dissected and mechanically homogenized whole hippocampus or HEK‐293 cells were lysed in an appropriate volume of RIPA lysis buffer (Beyotime, China) containing PMSF (YEASEN) for 1 h on ice and cleared by centrifugation for 10 min at 15,000 ***g*** at 4°C. Protein concentrations of the lysate were determined using Bradford reagent (Bio‐Rad, Hercules, CA, USA).

For western blot, tissue or cell lysates were separated by 10% SDS‐PAGE and transferred onto PVDF membrane (Biosharp). Membranes were then incubated with first antibodies, including PRG‐1 (1:3000; Synaptic System), GluR1(1:2000; CST), GluR2 (1:3000; Novus), GluN2A (1:2000; CST), GluN2B (1:2000; CST), NeuN (1:2000; CST), PSD‐95 (1:1500; CST), NSF (1:3000; Abcam), ß‐actin (1:5000; MP Biomedicals), washed, and then incubated with horseradish peroxidase (HRPO)‐conjugated secondary antibodies (1:5000; dianova). Finally, membranes were developed by enhanced chemiluminescence procedure (ECL, EpiZyme scientific). Quantification of immunosignals was performed using Image J 6.0 software (NIH, USA).

##### 4.3.2.7 Golgi‐Cox Staining and Dendritic Synapse Quantification

Golgi staining was used to examine neuroplasticity. After anesthesia, rat brains were removed and immediately stained by Hito Golgi‐Cox OptimStain Kit (Hitobiotec, USA) according to the manufacturer's instructions and then photographed under a microscope (Olympus DP80, Japan). Spines were defined as dendritic protrusions using Image J 6.0 software (NIH, USA) [62, 63]. In brain sections, the three areas of dendrites examined were: (1) the middle molecular layer of dentate gyri (moDG) granule cells; (2) stratum radiatum (sr, apical dendrites) and (3) stratum oriens (so, basal dendrites) of pyramidal neurons of the CA1 region. Adjust the threshold and set scale, then mark the spine region and width and automatically measure the area and width of the dendritic spine. Analysis was performed by researchers blinded to the treatment group.

##### 4.3.2.8 Enzyme‐Linked Immunosorbent Assay (ELISA) of Calcium Content

Hippocampal tissues in each group were homogenized in PBS buffer containing PMSF (YEASEN) at a general weight/volume (mg/μL) ratio of 1:9 using a homogenizer on ice. The homogenates were centrifuged at 5000 ***g*** for 10 min at 4°C, and the supernatant was collected to represent extracellular components. The precipitated cells were collected and washed three times carefully with PBS; then the cells were broken by ultrasonic method to represent intracellular components. Calcium was detected following the protocol of the calcium test kit (o‐cresolphthalein complexed copper colorimetric method) (Jianglai, China). The concentration of calcium was calculated according to the curve equation.

##### 4.3.2.9 CCK‐8 Assay

Primary hippocampal neurons from CON and RNS P3 rats were cultured in a 96‐well plate for 7 days in vivo. 10 μL CCK‐8 solution (Solarbio, China) was added to each well and incubated for 4 h in a 37°C incubator. The absorbance at 450 nm was measured using a microplate reader (Thermo Fisher, USA) to obtain the results.

##### 4.3.2.10 SOD Activity Assay

Primary hippocampal neurons from CON and RNS D3 rats were cultured for 7 days in vivo. The activity of SOD in cultured primary hippocampal neurons was measured with a SOD assay kit (vigorous, China) according to the manufacturer's instructions. The neurons were incubated with 2 μL DHE and 2 μL Hoechst33342 in a 37°C incubator for 30 min in the dark. They were washed and observed under a fluorescence microscope (Olympus DP80, Japan).

##### 4.3.2.11 Transmission Electron Microscopy

Rats were anesthetized with pentobarbital sodium (50 mg/kg body weight, i.p.) and the left ventricle was perfused with normal saline, followed by a mixture of 4% PFA and 2.5% GA (glutaraldehyde). Dissect the hippocampus and cut it into 1 mm × 1 mm × 1 mm. The sample was pre‐fixed with 2.5% glutaraldehyde and re‐fixed with 1% osmium tetroxide. Dehydrate step by step with acetone, permeation, and encapsulation by Epon‐812. Semi‐thin sections are observed under a light microscope, and the CA1 area of the hippocampus was selected. Then ultra‐thin sections of 60–90 nm were made by ultramicrotome (Leica UC7rt, Germany) and scooped onto copper wire. Staining was assayed by uranium acetate for 10–15 min, then lead citrate for 1–2 min. The JEM‐1400FLASH transmission electron microscope (JEOL, Japan) was used to collect images of copper mesh for the observation of the morphological structure of synapses in the hippocampal CA1 area.

##### 4.3.2.12 Hippocampal Brain Slice Preparation

Rats (P21–28 rats for data shown in Figures 8B,E–G and 9A–C of untreated CON and RNS, or P35–42 rats for data shown in Figures 8C,H–J and 9D–F of RNS with virus injection at P21) were anesthetized with 2% isoflurane, and then perfused with ice‐cold artificial cerebrospinal fluid (ACSF) (continuously oxygenated with 95% O_2_ + 5% CO_2_) containing (mM): 3 KCl, 25 NaHCO_3_, 1.25 NaH_2_PO_4_, 10 MgSO_4_, 0.5 CaCl_2_, 10 D‐Glucose, and 234 sucrose. After preparation, the pH of the solution was adjusted to 7.3–7.4 using concentrated hydrochloric acid. The brain was removed and coronal slices containing the hippocampus (265 μm thickness) were sectioned in chilled and oxygenated ACSF with a vibrating microtome (Leica, Germany), transferred to the slicing chamber for 1 h.

##### 4.3.2.13 In Vitro Electrophysiological Recording

For hippocampal neuron recordings, currents were measured under whole‐cell patch‐clamp recordings using pipettes with a typical resistance of 3–6 MΩ filled with internal solution containing (mM) 135 K‐MeSO_4_, 5 KCl, 0.5 CaCl_2_, 2 Mg‐ATP, 0.5 Na‐GTP, 5 EGTA, 5 HEPES, 10 Phosphocreatine, and 0.2% Biocytin, adjusted to a pH of 7.3 with an osmolarity of 290–300 mOsm/L. The external ACSF solution contained (in mM) 126 NaCl, 3 KCl, 26 NaHCO_3_, 1.25 NaH_2_PO_4_, 1 MgSO_4_, 1.2 CaCl_2_, and 10 D‐Glucose.

Cells were visualized with infrared optics on an upright microscope (BX51WI; Olympus). A MultiClamp 700B amplifier and pCLAMP10 software were used for electrophysiology (Axon Instruments). The series resistance and capacitance were compensated automatically after a stable Gigaseal was formed, with the membrane potential clamped at −70 mV. Spontaneous neuronal activity was recorded under current‐clamp (*I* = 0 pA) for 60 s consecutively. After allowing the cells to stabilize for 5 min, resting membrane potential (RMP) was determined during the silent period of neuronal spontaneous activity for 10 min [64]. Cells were stimulated with currents ranging from 0 to 100 pA (600 ms duration, 10 pA steps) and kept at rest. After allowing the cells to stabilize for 5 min, action potential (AP) was recorded under 60 pA stimulation [65].

Biocytin (Sigma, 5 mg/mL) was dissolved into the patch‐clamp pipette solution. After electrophysiological characterization, cells were held for at least 30 min in voltage clamp and constantly injected with a hyperpolarization current (500 ms, 50 pA, 0.5 Hz, 30 min) to allow biocytin filling (performed at 34°C). Spontaneous excitatory postsynaptic potentials (sEPSPs) in CA1 stratum radiatum were recorded for 10 min. In order to avoid bias by outlier values, outlier analysis was performed using GraphPad Prism (version 8.0.2; GraphPad Software Inc., USA) using the ROUT method, which is able to identify multiple outliers.

### 4.4 Quantification and Statistical Analysis

All experiments and data analyses were conducted in a blinded manner. Data were analyzed using GraphPad Prism (version 8.0.2; GraphPad Software Inc., USA). Quantitative data are expressed as the mean ± standard error of mean (SEM) throughout. All data underwent normality testing using the Kolmogorov–Smirnov Test. Statistical analysis was performed using a two‐tailed unpaired Student's *t*‐test for comparing two groups with normally distributed data or a Mann–Whitney *U* test for comparing two groups containing nonparametric distributed data. One‐way analysis of variance (ANOVA) with Bonferroni correction was used for comparing multiple groups containing normally distributed data or a Kruskal–Wallis test with a Dunn's multiple comparisons test for comparing multiple groups containing nonparametric distributed data. For comparison of multiple groups at a different time point, a two‐way ANOVA with repeated measures followed by Tukey's post hoc multiple comparisons test was used. Statistical significance was determined with an overall significance level of *p* < 0.05 (n.s. for *p* > 0.05, *p < 0.05, **p < 0.01, ***p < 0.001, ****p < 0.0001).

## Author Contributions

Conceptualization: X.L., Z.X., and S.C. Methodology: W.Z., Y.L., G.L., Q.L., and Z.S. Investigation: W.Z., Y.L., G.L., Q.L., Z.S., X.L., Z.X., and S.C. Writing – original draft: X.L. and W.Z. Writing – review and editing: Z.X. and S.C. Funding acquisition: X.L. Resources: X.L. Supervision: X.L. and Z.X. All authors contributed to drafting or editing parts of the manuscript and approved the final version of the manuscript.

## Conflicts of Interest

The authors declare no conflicts of interest.

## Supporting information


**Table S1:** cns70560‐sup‐0001‐TableS1.pdf.

## Data Availability

The data that support the findings of this study are available from the corresponding author upon reasonable request.
