# Supplementary material for: PRG‐1 Relieves Neonatal Stimuli‐Induced Hyperalgesia and Anxiety via Stage‐Specific Synapse Remodeling
Source: CNS Neurosci Ther. 2025 Aug 18;31(8):e70560. doi: 10.1111/cns.70560 (PMC12358805; doi:10.1111/cns.70560)
Supplement: Supplementary file 1 — Table S1: cns70560‐sup‐0001‐TableS1.pdf. [file CNS-31-e70560-s001.pdf]

Table S1. Grouping of rats (normally 6 rats for each experiment), related to STAR Methods

	groups	P3	3 weeks	6 weeks	9 weeks	12 weeks	P84+21	Total
first part	CON	CCK-8, ROS.	WB;<br>Golgi staining;<br>ELISA;<br>IF.	WB;<br>ELISA.	WB;<br>Golgi staining;<br>ELISA;<br>TEM;<br>IF.	WB;<br>ELISA.	TWL, MWT.	90 rats
	RNS	CCK-8, ROS.	WB;<br>Golgi staining;<br>ELISA;<br>IF.	WB;<br>ELISA.	WB;<br>Golgi staining;<br>ELISA;<br>TEM;<br>IF.	WB;<br>ELISA.	TWL, MWT.	90 rats
	IN						TWL, MWT.	6 rats
	RNS+IN						TWL, MWT.	6 rats
second part	CON		patch-clamp (10 rats)		WB;<br>ELISA.			22 rats
	RNS		patch-clamp (10 rats)		WB;<br>ELISA.			22 rats
	RNS + blank V			patch-clamp (10 rats)	behavioral test			16 rats
	RNS + PRG-1 OE			patch-clamp (10 rats)	WB;<br>ELISA.			22 rats
	RNS + PRG-1 SI			patch-clamp (10 rats)	WB;<br>ELISA.			22 rats
	RNS + ATPγS				WB;<br>ELISA.			12 rats
	RNS + ATP				WB;<br>ELISA.			12 rats
	RNS+verapamil				TWL, MWT.			6 rats
